# Molecular Co-Chaperone SGT1 Is Critical for Cell-to-Cell Movement and Systemic Infection of *Tomato Spotted Wilt Virus* in *Nicotiana benthamiana*

**DOI:** 10.3390/v10110647

**Published:** 2018-11-17

**Authors:** Xin Qian, Qing Xiang, Tongqing Yang, Hongyu Ma, Xin Shun Ding, Xiaorong Tao

**Affiliations:** Department of Plant Pathology, College of Plant Protection, Nanjing Agricultural University, Nanjing 210095, China; 2016202010@njau.edu.cn (X.Q.); 2016102033@njau.edu.cn (Q.X.); 2017102035@njau.edu.cn (T.Y.); mahongyu@njau.edu.cn (H.M.); xsdhome@hotmail.com (X.S.D.)

**Keywords:** molecular co-chaperone, *SGT1*, *Tomato spotted wilt virus*, TSWV NSm, tripartite negative stranded RNA virus

## Abstract

Tospovirus is a tripartite negative stranded RNA virus and is considered as one of the most devastating plant viruses. Successful virus infection in plant requires many host factors. To date, very few host factors have been identified as important in *Tospovirus* infection in plants. We reported earlier that NSm protein encoded by *Tomato spotted wilt virus* (TSWV), a type species of the genus *Orthotospovirus*, plays critical roles in viral cell-to-cell and long-distance movement. In this study, we determined that molecular co-chaperone NbSGT1 interacted with TSWV NSm in *Nicotiana benthamiana*. TSWV infection significantly upregulated the expression of *NbSGT1* gene and transient overexpression of *NbSGT1* in *N. benthamiana* leaves accelerated TSWV infection. In contrast, silencing the *NbSGT1* gene expression using a virus-induced gene silencing (VIGS) approach strongly inhibited TSWV NSm cell-to-cell movement, as well as TSWV local and systemic infection in *N. benthamiana* plants. Furthermore, NbSGT1 was found to regulate the infection of both American and Euro/Asia type tospoviruses in *N. benthamiana* plant. Collectively, our findings presented in this paper and the results published previously indicated that molecular co-chaperone NbSGT1 plays important roles in modulating both positive stranded and tripartite negative stranded RNA virus infection in plants.

## 1. Introduction

Host chaperone proteins are critical for infection of both animal and plant viruses. Heat shock protein 70 (Hsp70) and heat shock protein 90 (Hsp90) are two most commonly identified host chaperone proteins involved in virus infection. Hsp70 interacts with the replicase of Tombusvirus or Tymovirus [1,2], the coat protein of Potyvirus [3], and plays important roles during virus infections. Closteroviruses also encode an Hsp70 homolog important for virus intercellular movement [4,5]. Hsp70 and Hsp90 are also involved in the assembly of the replicase complex of Red clover necrotic mosaic virus [6] and in the establishment of Tomato yellow leaf curl virus infection in plant [7]. Suppressor of the G2 allele of skp1 (SGT1) is known as a co-chaperone that interacts with Hsp70 [8] and form a SGT1-Hsp70 chaperone complex which can regulate Arabidopsis immune responses [9]. SGT1 can interact with Hsp90 and required for Mla12 resistance 1 (RAR1) to form an Hsp90-SGT1-RAR1 complex to modulate the innate immune response in plants [10,11,12]. SGT1 is also known to regulate the protein accumulation of nucleotide binding-leucine-rich repeat (NLR) immune receptors [11], to control the intramolecular domain interactions to maintain NLR receptors in inactive states [13], and to participate in the nuclear and cytoplasmic distribution of tobacco N NLR [14]. SGT1 is also required for host and nonhost disease resistance [15]. In addition, SGT1 was reported to regulate the infection of Potato virus X (PVX), a positive stranded RNA virus, in its host plants [16]. Whether SGT1 could regulate negative stranded RNA virus infection in host plants was un-explored before this study.

*Tomato spotted wilt virus* (TSWV) is the type species of the genus *Orthotospovirus* and infects a wide range of agronomic and ornamental crops [17,18,19,20]. TSWV is considered as one of the most devastating plant viruses, causing about one billion dollars crop loss annually worldwide [21,22]. The virus is transmitted by thrips in a persistent propagative manner [23]. The TSWV has tripartite negative stranded RNA segments (e.g., L, M and S) [23,24]. The L segment encodes an RNA-dependent RNA polymerase (RdRP) in the antisense strand [25,26]. The M segment encodes a movement protein (NSm) in the sense orientation and a glycoprotein in the antisense orientation which is further processed into two mature Gn and Gc proteins [27,28]. The S segment encodes a non-structural protein (NSs) acting as an RNA silencing suppressor [29,30] in the sense strand, and a nucleocapsid protein, in the antisense strand.

TSWV NSm plays a critical role in virus cell-to-cell movement and systemic infection. The NSm protein was shown to localize on the endoplasmic reticulum (ER) membrane and plasmodesmata (PD) in plant cell walls [31,32,33], to modify PD for virus trafficking between cells [33,34], and to bind viral RNA [35]. Expression of TSWV NSm in *N. rustica* protoplasts resulted in the formation of tubular like structures [36,37]. Heterologously expressed NSm supported the local and long-distance movement of a movement deficient mutant of Tobacco mosaic virus (TMV) [36,38]. Although TSWV NSm was reported to interact with tobacco DnaJ and Arabidopsis At4-1 [35,39], the functions of these protein-protein interactions remained unknown.

Virus relies on cellular factors to establish infection in its host, however, very few host cellular factors required for TSWV infection were identified. In this study, we determined that host molecular co-chaperone NbSGT1 associated with TSWV NSm and this interaction was found to facilitate TSWV cell-to-cell movement and systemic infection in plant. Transient overexpression of NbSGT1 in *N. benthamiana* leaves promoted TSWV infection in this host plant. In contrast, silencing the NbSGT1 gene expression using a Tobacco rattle virus (TRV) -based virus-induced gene silencing (VIGS) strongly inhibited TSWV local and systemic infection in *N. benthamiana* plants. NbSGT1 also regulates the infection of INSV from American type tospoviruses and TZSV from Euro/Asia type tospoviruses in plant. We conclude that NbSGT1 plays important roles in tospovirus infection in plants.

## 2. Materials and Methods

### 2.1. Host Plant and Virus

Six- to eight-week old *N. benthamiana* plants were used for transient expression and virus inoculation assays. Source of TSWV Yunnan isolate was described previously [40]. TZSV and INSV isolates were collected from Yunnan province, and maintained in this laboratory. TSWV Yunnan-, TZSV- or INSV-infected *N. benthamiana* systemic leaves were harvested and homogenized in a 0.01 M phosphate buffer (PB), pH 7.4, and the crude leaf extracts were rub-inoculated to leaves of *N. benthamiana* plants. The inoculated plants were grown inside a growth chamber set at 24 °C with a 16 h light/8 h dark photoperiod.

### 2.2. Construction of Expression Vectors

TSWV NSm gene was PCR amplified respectively using specific primers, and fused with a FLAG or a YFP tag at its C-terminus. NbSGT1 from *N. benthamiana* was fused with hemagglutinin (HA) at C-terminus. The ligated fragments were cloned individually behind a duplicated 35S promoter in the binary vector p2300S [41]. For bimolecular fluorescence complementation assay (BiFC), NbSGT1 was fused to the N-terminal half of a YFP on pCV-nYFP while the NSm was fused to the C-terminal half of the YFP on pCV-cYFP. The expression vector mCherry-HDEL//NSm-GFP harboring both NSm-GFP and mCherry-HDEL expression cassettes was described previously [31]. To silence NbSGT1 gene in *N. benthamiana* plants, the 625 nucleotides of NbSGT1 gene was PCR amplified and inserted into the TRV2 vector as instructed [15]. The same TRV vector carrying a fragment of 300 nucleotides from a GUS gene was used as a control vector. All the primers used in this study are listed in Table S1.

### 2.3. Agrobacterium-Mediated Transient Expression and Bimolecular Fluorescence Complementation (BiFC) Assays

Expression vectors were individually electroporated into Agrobacterium strain GV3101 cells. The transformed Agrobacterium cells were cultured for 24 h and then pelleted by 10 min centrifugation at 6600 rpm. The resulting pellets were individually resuspended in an agro-infiltration buffer containing 100 µM acetosyringone, and incubated for 3–5 h at 28 °C. Fully expended *N. benthamiana* leaves were infiltrated with various Agrobacterium cultures (OD_600_ = 0.25 or diluted 500 times for cell-to-cell movement assay) using needle-less syringes. For BiFC assay, the infiltrated leaves were harvested at 24 h post agro-infiltration (hpai) and then examined for fluorescence signals under a Carl Zeiss LSM 710 confocal microscope (Carl Zeiss, Jena, Germany).

### 2.4. Western Blot and Co-Immunoprecipitation (co-IP) Assays

Agro-infiltrated leaves were harvested and used for co-IP assays. The harvested leaf tissues were homogenized in liquid nitrogen and then resuspended in an extraction buffer containing 10% glycerol, 25 mM Tris-HCl, pH 7.5, 1 mM EDTA, 150 mM NaCl, 2% Polyvinylpolypyrrolidone (PVPP), 10 mM DTT, 1× Protease inhibitor cocktail (Sigma, Shanghai, China), 0.2% TritonX-100 (Sigma-Aldrich, St. Louis, MO, USA) (1 g tissue per sample/2 mL buffer). Plant crude extracts were centrifuged twice at 12,600× *g* for 10 min each time, at 4 °C. Each supernatant (500 μL) was mixed with 45 μL anti-HA or anti-FLAG conjugated agarose beads (Sigma) and incubated at 4 °C for 1.5 h. Agarose beads were pelleted and washed six times with the co-IP buffer (10% glycerol, 25 mM Tris-HCl, pH 7.5, 1 mM EDTA, 150 mM NaCl, 2% PVPP, 1 mM DTT, 0.1% Triton X-100). The resulting pellets were mixed individually with 30 μL 3× SDS loading buffer (150 mM Tris-HCl, pH 6.8, 6% SDS, 0.3% Bromophenol blue, 30% glycerol, 300 mM DTT) and boiled for 8 min. For immunoblot, proteins were separated in 10% SDS-PAGE gels through electrophoresis, and then transferred to PVDF membranes. The blots were probed with an anti-HA, anti-YFP or anti-TSWV N antibody followed by an HRP-conjugated secondary antibody. The detection signals were developed using an ECL reagent as instructed (Thermo Scientific, Hudson, NH, USA), and visualized using a Bio-Rad ChemiDoc Touch imaging system (Bio-Rad, Hercules, CA, USA). For Mass Spectrometry analysis, the Co-IPed products was silver stained in an SDS-PAGE gel. The specific band was cut out and analyzed by Matrix-Assisted Laser Desorption/Ionization Time of Flight Mass Spectrometry (MALDI-TOF-MS, Bruker Daltonic Inc., Billerica, MA, USA) in core facility of College of Plant Protection in Nanjing Agricultural University.

### 2.5. Quantitative RT-PCR (qRT-PCR)

Total RNA was extracted from the assayed *N. benthamiana* leaf tissues using a total RNA isolation kit as instructed (Tiangen, Beijing, China), different samples from different plants. For first strand cDNA synthesis, 1 μg total RNA was used in each 20 μL reaction using an Oligo-dT primer and a PrimeScrip RT reagent kit (Takara, Dalian, China), followed by qPCR on a Bio-Rad CFX ConnectTM Real-Time PCR system using a Power SYBR Green Master Mix (Life Technologies, Carlsbad, CA, USA). The Nb-β Actin and Nb-EF1a genes were used as internal controls. All the primers used for RT-PCR are listed in Table S1. The resulting qPCR data were analyzed as previously described [42].

## 3. Results

### 3.1. TSWV NSm Interacts with NbSGT1

**Figure 1:**
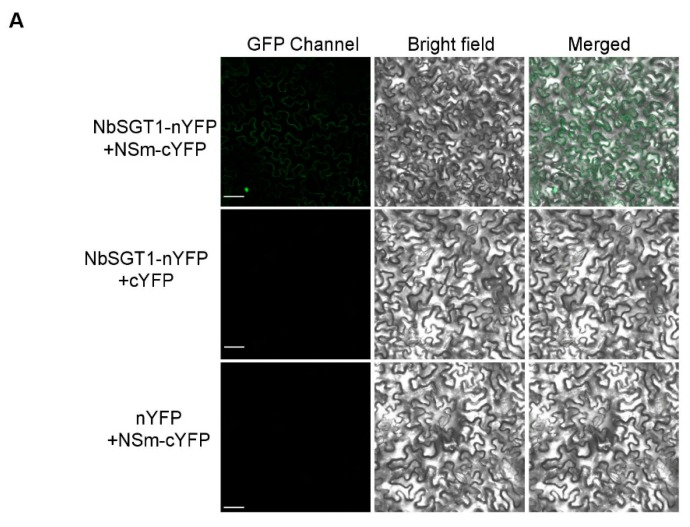

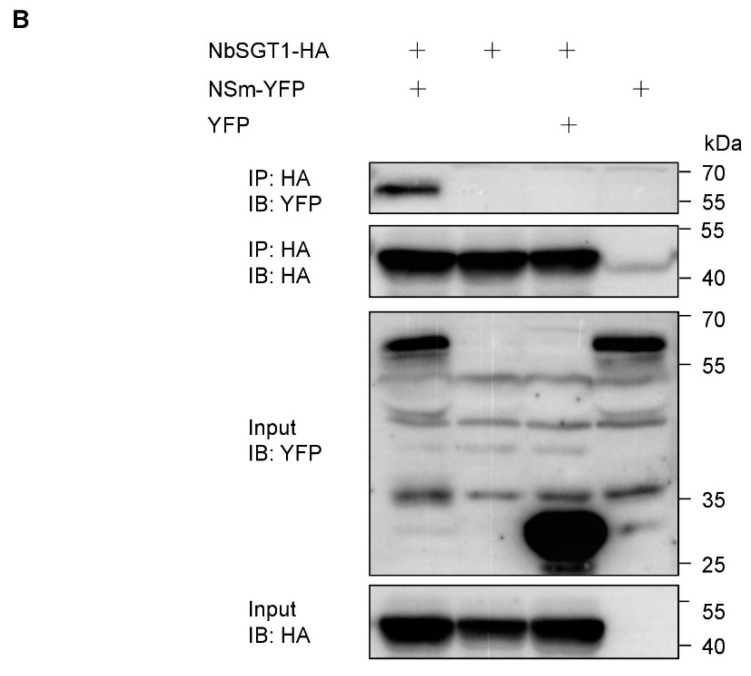
NbSGT1 interacts with TSWV NSm in *Nicotiana benthamiana* leaf cells. (**A**) BiFC assay of the interaction between NbSGT1 and TSWV NSm in *N. benthamiana*. NbSGT1–nYFP and NSm–cYFP, NbSGT1–nYFP and cYFP or nYFP and NSm–cYFP were co-expressed in *N. benthamiana* leaves. The fluorescent signals were detected by a confocal microscope at 24 h post agro-infiltration. Scale bars = 100 μm. (**B**) Co-immunoprecipitation analysis of the interaction between NbSGT1 and NSm. The YFP was fused at C-terminus of NSm (NSm-YFP) and transiently co-expressed with the NbSGT1-HA in *N. benthamiana* leaf tissues. The SGT1-HA was used to co-immunoprecipitate NSm-YFP. The blots were probed with a GFP specific multiclonal antibody or a HA specific monoclonal antibody. IB, immunoblot with specific antibody; IP, immunoprecipitation with specific antibody. The sizes of proteins in kDa are shown left. Ponceau S staining was used to show the protein loading.

To identify host factor(s) involving in TSWV cell-to-cell movement and systemic infection in *N. benthamiana*, we transiently expressed TSWV NSm-FLAG fusion in *N. benthamiana* leaves and then immunoprecipitated this fusion protein from leaf crude extract using a FLAG-trap agarose. The TSWV NSm without FLAG TAG was used as a negative control. Immunoblot analysis showed that the NSm-FLAG protein was retained on the column (Figure S1A). The co-IPed product was also separated on SDS-PAGE and silver stained. An extra band about 45 kDa in NSm-FLAG sample compared to NSm without FLAG (Figure S1B) in the silver stained gel was cut out and analyzed by MALDI-TOF-MS. The result of the assay showed that SGT1 was immunoprecipitated by the NSm-FLAG. *N. benthamiana* contains SGT1.1 and SGT1.2 [15] and two NbSGT1 homologs have 99.7% sequence identity. To further confirm the interaction between NSm and SGT1 proteins, we cloned SGT1.2 (refer to NbSGT1 hereafter) from *N. benthamiana* and fused NbSGT1 to the N-terminal half of the YFP (nYFP) and also fused NSm to the C-terminal half of the YFP (cYFP). These two fusion proteins were co-expressed in *N. benthamiana* leaves through agro-infiltration, and the infiltrated leaves were harvested at 24 h post agro-infiltration (hpai) for BiFC analysis. Results shown in Figure 1A demonstrated that co-expression of NbSGT1-nYFP and NSm-cYFP in same *N. benthamiana* leaves resulted in a strong yellow fluorescence. Co-expression of NbSGT1-nYFP and cYFP, or nYFP and NSm-cYFP control in *N. benthamiana* leaves, however, did not produce any yellow fluorescence signals in the infiltrated leaves.

We next conducted a co-immunoprecipitation assay to further investigate the interaction between NbSGT1 and NSm. NbSGT1 was fused to a HA tag at its C-terminus. The SGT1-HA and NSm-YFP fusion were co-expressed in *N. benthamiana* leaves through agro-infiltration. At 24 hpai, the infiltrated leaves were harvested and used for the immunoprecipitation assays. Results from the assays showed that SGT1-HA was indeed co-immunoprecipitated with NSm-YFP. This co-immunoprecipitation result was not observed when the leaves were co-infiltrated with SGT1-HA and YFP or infiltrated with NSm-YFP alone (Figure 1B).

### 3.2. TSWV Infection in N. benthamiana Up-Regulates NbSGT1 Expression and Overexpression of NbSGT1 Enhances TSWV Infection in Plant

**Figure 2:**
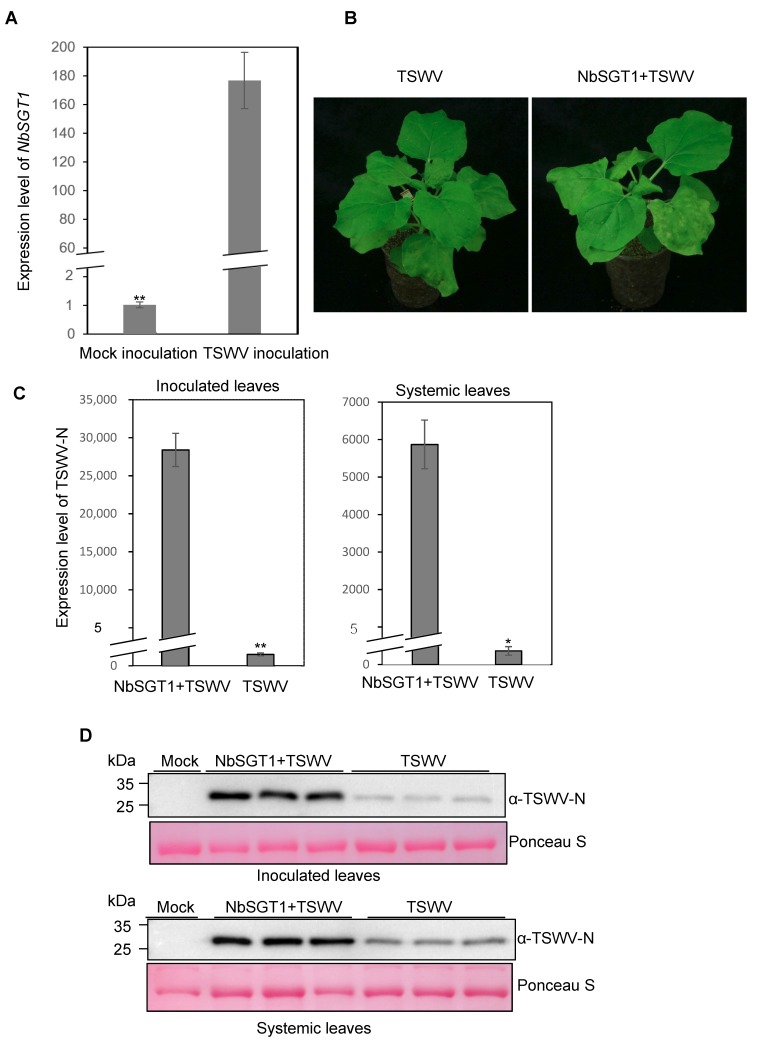
Up-regulation of *NbSGT1* expression promoted virus infection. (**A**) *NbSGT1* RNA levels were significantly up-regulated after TSWV infection. qRT–PCR was used to determine the levels of *NbSGT1* RNA transcripts in the TSWV-inoculated or mock-inoculated *N. benthamiana* plants. The mock-inoculated plants were used as controls. The error bars indicate standard deviation (SD) (*n* = 3) (**B**) Transient over-expression of NbSGT1 promoted TSWV infection in *N. benthamiana*. At one day post transient over-expression of NbSGT1, the infiltrated leaves were inoculated with a crude sap from TSWV-infected leaf tissues. The inoculated plants were photographed at 7 dpi. Plants agro-infiltrated with an *Agrobacterium* carrying the empty expression vector and then inoculated with TSWV were used as controls. (**C**) The quantitative RT-PCR analysis of TSWV N expression in the inoculated and systemically infected leaves harvested from the NbSGT1-overexpressed or non-over-expressed plants. The error bars represent SD (*n* = 3), * *p* < 0.05, ** *p* < 0.01. (**D**) Immunoblot analysis of TSWV accumulation in the NbSGT1-over-expressed or non-over-expressed *N. benthamiana* plants. The accumulation of TSWV N in the inoculated leaves was determined at 5 dpi, and in the systemic leaves at 7 dpi using a TSWV N specific antibody. Ponceau S staining was used to show the protein loadings.

To determine the response of NbSGT1 to TSWV infection, we isolated total RNA from leaves of the TSWV- or mock-inoculated *N. benthamiana* plants. qRT-PCR results showed that the expression level of NbSGT1 was strongly up-regulated in the TSWV-infected plants (Figure 2A). This finding prompted us to transiently overexpress NbSGT1 in *N. benthamiana* leaves through agro-infiltration. At 24 hpai of the leaves infiltrated with an Agrobacterium carrying the p1300-NbSGT1-HA, the infiltrated leaves were inoculated with crude extracts from TSWV-infected leaves. Leaves infiltrated with an Agrobacterium carrying an empty vector and then inoculated with TSWV-infected crude extract were used as controls. Result showed that transiently expression of NbSGT1 significantly enhances TSWV infection in *N. benthamiana* (Figure 2B). qRT-PCR results showed that the accumulation of TSWV N mRNA in NbSGT1- overexpressed plants was significantly increased in both inoculated and systemic leaves compared to those in non-overexpressed plants at 7 days post-inoculation (dpi) (Figure 2C). Western blot assay showed that more TSWV structural nucleocapsid (N) protein was accumulated in the inoculated leaves transient overexpressing NbSGT1-HA than that in the control plant leaves (Figure 2D, upper panel). Similar results were also observed in the systemically infected leaves (Figure 2D, lower panel). To ask whether the infiltrated NbSGT1-HA protein is moving through the vascular tissue, we examined the accumulation of NbSGT1 in systemic leaves of *N. benthamiana*. The result showed that the NbSGT1-HA was not detected in systemic leaves (Figure S1C).

### 3.3. Silencing NbSGT1 Expression through VIGS Inhibits TSWV NSm Intercellular Movement

**Figure 3:**
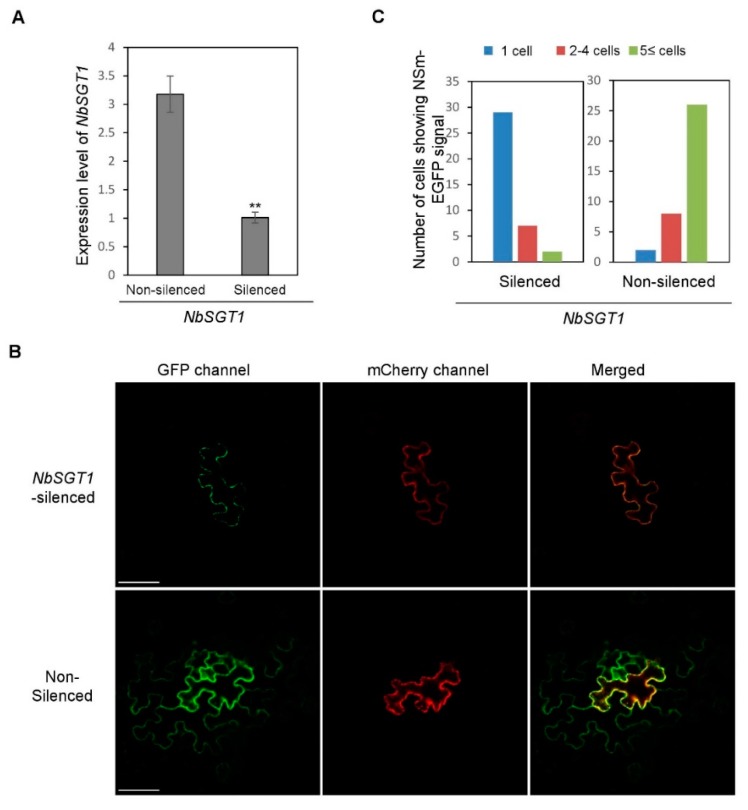
Silencing NbSGT1 gene expression strongly inhibited TSWV cell-to-cell movement. (**A**) Quantitative RT-PCR analysis of NbSGT1 expression silenced by TRV-mediated VIGS in *N. benthamiana*. The error bars represent SD (*n* = 4), ** refers to *p* < 0.01. (**B**) Silencing NbSGT1 gene expression through VIGS inhibited TSWV NSm cell-to-cell movement in *N. benthamiana* leaves. NbSGT1 expression was silenced using a TRV-based VIGS vector. The TSWV NSm cell-to-cell movement was examined after infiltrating an Agrobacterium carrying the construct mCherry-HDEL//NSm-GFP into the NbSGT1-silenced or non-silenced control plants. The number of cells in each fluorescent foci was counted under a confocal microscope. Bars = 50 μm. (**C**) Analysis of TSWV NSm-GFP cell-to-cell movement in the NbSGT1-silenced or non-silenced *N. benthamiana* plant leaves.

TSWV NSm-YFP fusion is known to move effectively between cells of its host plants [31]. To determine if silencing NbSGT1 expression would impact NSm movement between *N. benthamiana* leaf cells, we used a previously described strategy to silence NbSGT1 in *N. benthamiana* plant leaves with the TRV-based VIGS vector carrying a fragment of the NbSGT1 gene [15] or used TRV with a fragment of the GUS gene as negative control. At two weeks post TRV infection, the systemic leaves of plants were analyzed for NbSGT1 expression by quantitative RT-PCR. The result of the assay showed that the expression level of NbSGT1 was knocked down significantly compared with that in the control plants (Figure 3A). The systemic leaf of NbSGT1-silenced and non-silenced plants were then infiltrated with an Agrobacterium carrying the construct mCherry-HDEL//NSm-GFP co-expressing a mCherry-HDEL fusion and an NSm-EGFP fusion [31]. Under the confocal microscope, mCherry-HDEL containing an ER retention signal was anchored on the ER membrane and restricted in the infiltrated single cell (Figure 3B, upper panel), while the NSm-EGFP fusion moved from one cell to another (Figure 3B, lower panel). The result also showed that the cell-to-cell movement of NSm-GFP fusion was significantly impeded in the plants silenced for NbSGT1 expression (Figure 3B,C; Table S2). Similar results were obtained in other two repeated experiments.

### 3.4. Silencing NbSGT1 Expression Inhibits TSWV Local and Systemic Infection in N. benthamiana

**Figure 4:**
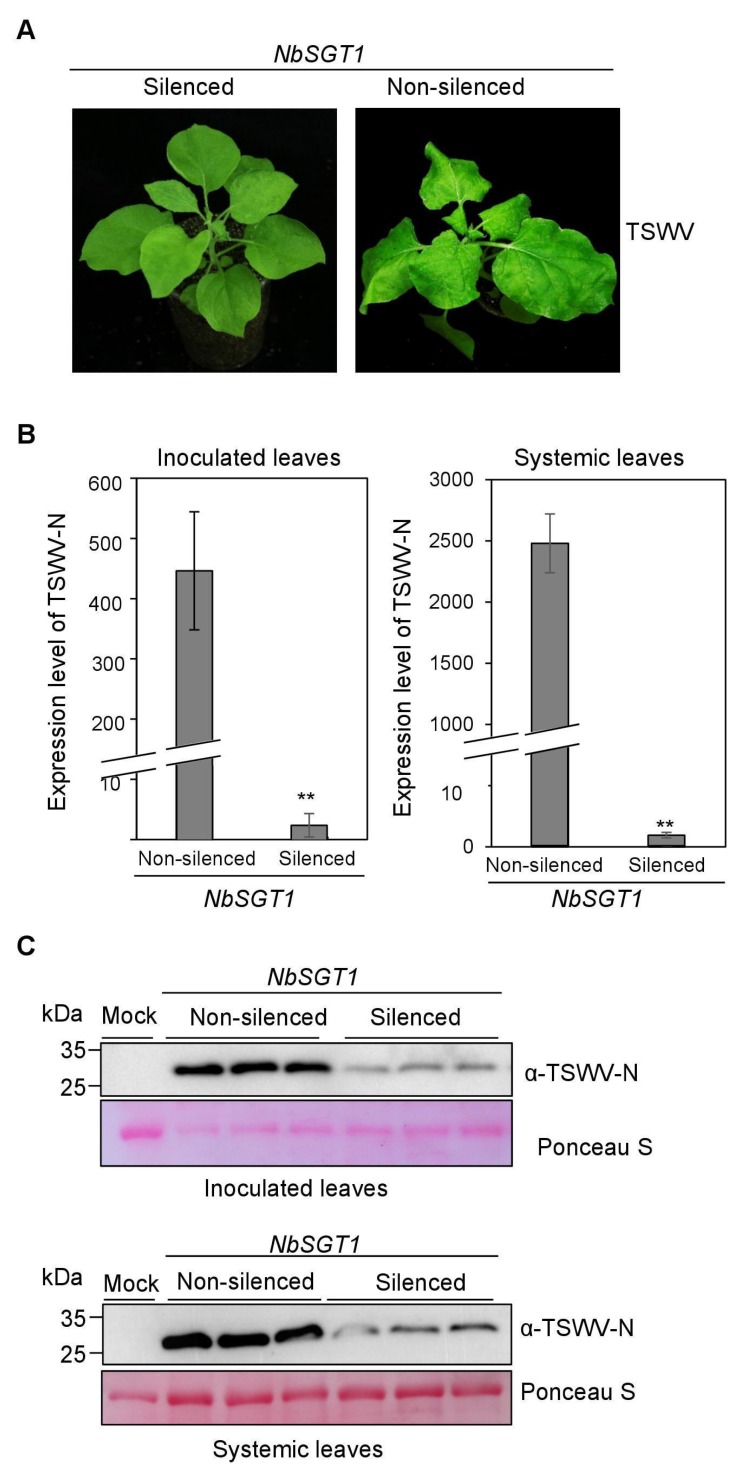
Silencing *NbSGT1* gene expression through VIGS significantly inhibited TSWV local and systemic infection in *N. benthamiana*. (**A**) The NbSGT1-silenced or non-silenced plants were inoculated with crude saps from TSWV-infected leaf tissues. The inoculated plants were photographed at 7 dpi. (**B**) The quantitative RT-PCR analysis of TSWV N expression in the inoculated and systemically infected leaves harvested from the NbSGT1-silenced or non-silenced plants. The *actin* was used as the internal reference gene. The error bars represent SD (*n* = 4), ** *p* < 0.01. (**C**) Immunoblot analysis of TSWV N protein accumulation in the inoculated and systemically infected leaves harvested from the NbSGT1-silenced or non-silenced plants. The inoculation leaves were harvested and analyzed at 5 dpi, and the systemic leaves were harvested and analyzed at 7 dpi.

The NbSGT1-silenced and non-silenced *N. benthamiana* plants were also inoculated with crude saps from TSWV-infected plants. The upset of TSWV symptoms in the NbSGT1-silenced plants was delayed by about five days compared with that in the non-silenced plants (Figure 4A). qRT-PCR results showed that the accumulation of TSWV N mRNA in NbSGT1-silenced plants was significantly reduced in both inoculated and systemic leaves compared to those in non-silenced plants at 7 dpi (Figure 4B and Figure S4A). Western blot results further confirmed that the accumulation of TSWV N protein was significantly reduced in the inoculated and systemic leaves of the NbSGT1-silenced plants (Figure 4C). This experiment was repeated three times and similar results were obtained. To test if TRV has any effects on the accumulation of TSWV, the expression of TSWV N RNA was examined in both TRV-GUS infected plants and non-infected plants, the results indicate that the accumulation of TSWV has no significant difference between TRV-infected plants and non-infected plants (Figure S2A,B). To examine whether the silencing of NbSGT1 gene was maintained in TSWV inoculated leaves at 7 days post TSWV inoculation on TRV-SGT1 infected plants, we further analyzed the expression of the NbSGT1 gene in the TSWV inoculated leaves of both TRV-GUS infected plants and TRV-SGT1 infected plants. qRT-PCR analyses showed that the NbSGT1 gene was still silenced in TSWV inoculated leaves at 7 dpi (Figure S3A,B).

### 3.5. NbSGT1 Regulates Both American and Euro/Asia Type Tospovirus Infection in N. benthamiana

**Figure 5:**
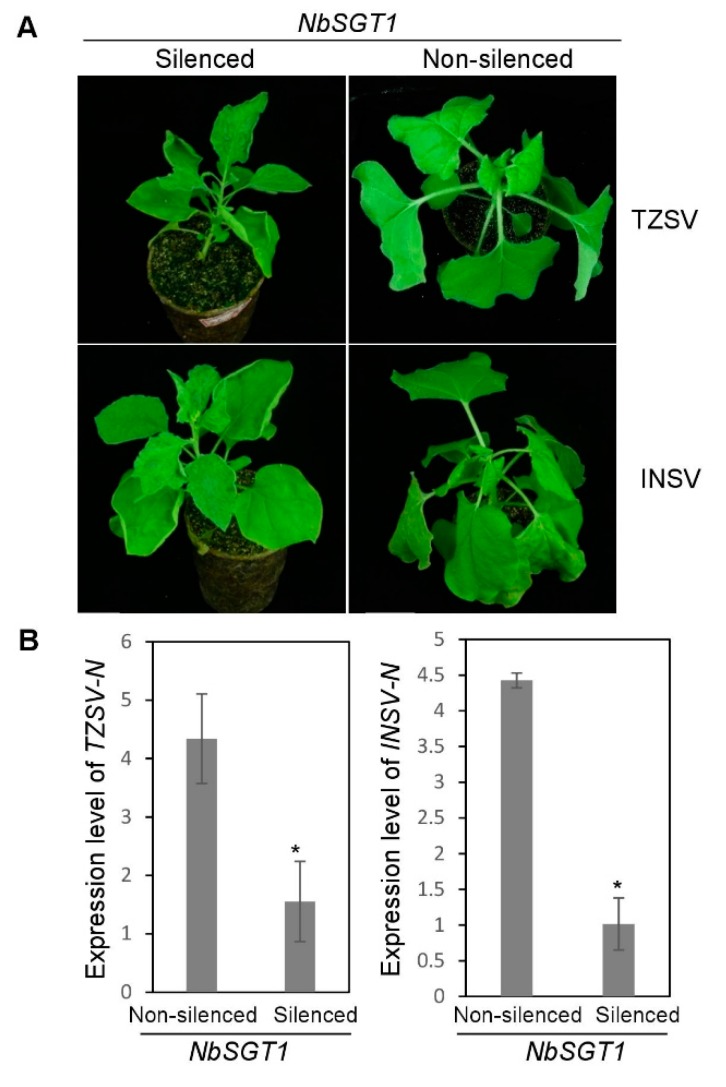

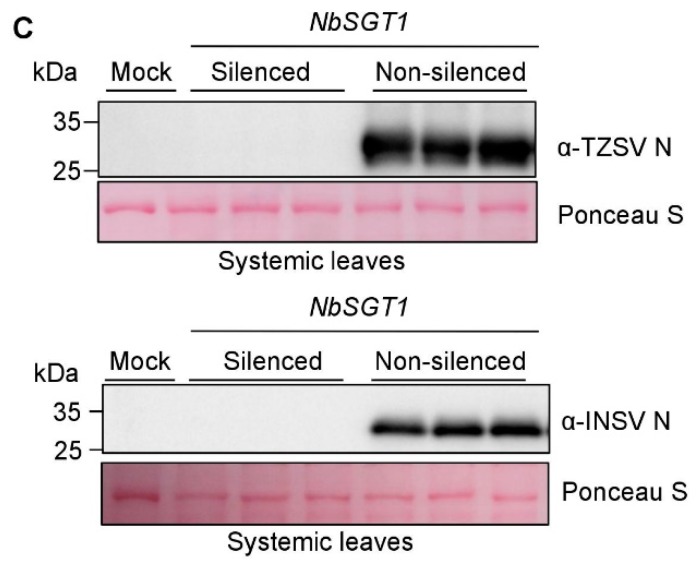
The effect of NbSGT1 on TZSV or INSV infection in *N. benthamiana* plants. (**A**) *N. benthamiana* plants were silenced for NbSGT1 gene expression through VIGS. The NbSGT1-silenced plants were inoculated with TZSV or INSV. The non-silenced *N. benthamiana* plants were also inoculated with one of the two viruses and used as controls. The virus-inoculated plants were photographed at 10 dpi. (**B**) The quantitative RT-PCR analysis of TZSV N or INSV N expression in the systemically infected leaves harvested from the NbSGT1-silenced or non-silenced plants. The *actin* was used as internal reference gene. The error bars represent SD (*n* = 4), * refers to *p* < 0.05. (**C**) Immunoblot analysis of TZSV and INSV N protein accumulations in the systemically infected leaves harvested from the NbSGT1-silenced or non-silenced plants at 10 dpi, using an anti-TZSV N or an anti-INSV N antibody. Ponceau S staining was used to show the protein loadings.

The genus *Orthotospovirus* currently include more than twenty recognized and tentative species. According to the geographic distributions and nucleocapsid sequence homology, the members of the genus Orthotospovirus can be further divided into the American and the Euro/Asian type tospoviruses [24]. Next, we inoculated NbSGT1-silenced or non-silenced *N. benthamiana* plants with tomato zonate spot virus (TZSV, an Euro/Asian type tospovirus) or Impatient necrosis spot virus (INSV, an American type tospovirus) as described for TSWV above. Although the NbSGT1-silenced plants did show somewhat reduced growth and leaf curling, TZSV infection symptoms were observed by fourteen dpi. On the non-silenced control plants, TZSV symptoms were observed by nine dpi (Figure 5A, upper panel). Virus symptoms were observed on the INSV-inoculated NbSGT1-silenced plants by eleven dpi, and on the INSV-inoculated non-silenced plants by seven dpi (Figure 5A, lower panel). The accumulation of INSV or TZSV N mRNA in NbSGT1-silenced plants was significantly lower than that in non-silenced plants (Figure 5B and Figure S4B,C). Western blot results showed that the viral N protein accumulated much less in the TZSV- or INSV-inoculated NbSGT1-silenced plants compared with that in the virus-inoculated non-silenced control plants (Figure 5C). In both virus infection experiments, qRT-PCR results confirmed the reduced expression of the NbSGT1 mRNA in TRV-SGT1 plants at 14 dpi and the inoculated leaves at additional 7 dpi (Figure S3C–F).

## 4. Discussion

The previous studies have shown that the movement protein NSm protein of TSWV plays an important role in TSWV cell-to-cell movement [27,28]. In this study, we obtained evidence showing that molecular co-chaperone NbSGT1 interacted with TSWV NSm, based on the co-immunoprecipitation and BiFC assay in *N. benthamiana* leaf cells, and this interaction is important for the NSm movement function. Transient overexpression of NbSGT1 in local *N. benthamiana* leaves promoted the cell-to-cell and systemic movement of TSWV. In contrast, silencing the NbSGT1 expression in *N. benthamiana* plants strongly inhibited TSWV local and systemic infection. In addition, NbSGT1 also regulate the infection of INSV from American tospoviruses and the infection of TZSV from Euro/Asia type tospoviruses in *N. benthamiana*.

NbSGT1 is known to play a positive role in host resistance response mediated by NLR proteins [15]. Silencing of NbSGT1 compromised N-, Rx-, and Pto NLR-mediated resistance to Tobacco mosaic virus (TMV), PVX and *Pseudomonas syringae* pv. tabaci (avrPto). NbSGT1 is also required for nonhost disease resistance in plants [15]. Silencing of NbSGT1 gene expression in *N. benthamiana* also significantly reduced the nonhost resistance to *P. syringae* pv. maculicola, *Xanthomonas axonopodis* pv. vesicatoria and *X. campestris* pv. campestris [15]. In contrast to the role of SGT1 in host and nonhost disease resistance against pathogen invasions, we demonstrated that NbSGT1 plays a positive role in tospovirus infection. Consistent with our findings, NbSGT1 was also reported to play a positive role in PVX infection in *N. benthamiana* [16]. PVX infection strongly up-regulated NbSGT1 expression and this gene upregulation lead to a higher level of PVX accumulation in *N. benthamiana* plants [16]. In the same report the authors also showed that overexpression of PVX TGBp3 alone could up-regulate NbSGT1 expression. Because PVX TGBp3 is known as a movement protein, we speculate that the up-regulation of NbSGT1 expression by PVX TGBp3 expression may be responsible for accelerating PVX movement in *N. benthamiana* [16]. However, it remains unknown if NbSGT1 directly associates with PVX TGBp3. In this study, we found that the TSWV infection in *N. benthamiana* significantly upregulated the expression of NbSGT1 gene. Silencing of NbSGT1 gene expression strongly inhibited cell-to-cell movement of TSWV NSm-GFP and impeded the systemic infection. Taken together, although SGT1 is required for host and non-host resistance, molecular co-chaperone SGT1 also plays an important positive role in the infection of PVX and TSWV, a positive and negative stranded RNA virus, respectively.

SGT1 was also shown to regulate the nucleocytoplasmic partitioning of tobacco N NLR immune receptor [14]. Our findings that SGT1 functions in NSm movement suggest that SGT1 may not only involve in modulating intracellular trafficking of certain cellular proteins but also have an important role in the viral intercellular movement. TSWV NSm was shown to target into PD and move by itself from cell to cell [31]. Tobacco DNAJ were previously found to interact with TSWV NSm in a yeast two-hybrid screening assay [35]. Although the function of DNAJ in the viral movement of TSWV NSm remains unknown, Du and coworkers reported that *N. benthamiana* DNAJ also associated with tobamovirus MP. Importantly, the DNAJ is required for TMV infection [43]. DNAJ is known to interact with Hsp70 or with Hsp90 and RAR1 to form co-chaperon complexes [9,10]. Interestingly, the closterovirus itself encodes a viral Hsp70 protein which targets to PD and this viral Hsp70 plays a critical role in cell-to-cell movement of closterovirus [5]. Chaperone protein Hsp90 and Hsp70 were reported to deliver Tom70, an import receptor, to mitochondria [44]. As Hsp90 or Hsp70 associates with SGT1 and forms chaperone/co-chaperone complex, it is possible that these chaperone/co-chaperone complex may help to deliver viral movement protein to PD or help to unfold and refold viral movement and replication complexes to facilitate virus trafficking between plant cells through PD.

eIF4E and its isoform eIF(iso)4E carrying a mutation have been shown to confer resistance to many plant viruses, including Potyvirus, Cucumber mosaic virus (CMV), Turnip crinkle virus (TCV) and etc. [45]. Several host factors or host machineries involved in TSWV replication, transcription and movement have been characterized. Tobacco eEF1A was reported to enhance both replication and transcription of TSWV in vitro [46]. TSWV N protein was found to associate with actin filaments and traffic along the actin network, and treatment with the actin depolymerizing drug latrunculin B (LatB) strongly inhibited the TSWV infection in tobacco plant [47]. The Arabidopsis RHD3 gene was also shown to involve in TSWV movement. The cell-to-cell movement and systemic infection of TSWV was significantly delayed in rhd3 mutant that disrupted in ER network [31]. The NbSGT1 characterized in this study involved in local movement and systemic infection of TSWV in *N. benthamiana* plant. Because clustered regularly interspaced short palindromic repeats (CRISPR) -Cas9 has become a powerful gene editing technology [48,49,50], these host factors or host machineries may be potential candidates to be mutagenized and modified in crops to confer resistance to tospoviruses.

## 5. Conclusions

In summary, we demonstrated in this study that a molecular co-chaperone NbSGT1 interacts with TSWV NSm and plays an important role in cell-to-cell movement and systemic infection of TSWV in *N. benthamiana*. INSV and TZSV from both American and Euro/Asia type tospoviruses in the genus Orthotospovirus also require the NbSGT1 to establish a robust local viral accumulation and systemic infection in *N. benthamiana*. Hence, SGT1 positively regulates negative stranded RNA virus infection in plants. Our results also suggested that SGT1 functions as a susceptible host factor for tospovirus infection. As susceptible host factors are good candidates that can be genetically modified for resistance crops breeding against virus infection, SGT1 offers an important genetic resource to be edited by new gene technologies in crops to control tospovirus diseases.

## Supplementary Materials

The following are available online at http://www.mdpi.com/1999-4915/10/11/647/s1. Figure S1: Analysis of Co-IPed NSm-FLAG in SDS–PAGE. (A and B) Co-IPed NSm-FLAG products were analyzed by immunoblot (A) and silver stained SDS–PAGE analysis (B). NSm without FLAG was used as negative control. IB, immunoblot with specific antibody; IP, immunoprecipitation with specific antibody. The protein size markers are shown in the left. (C) Immunoblot analysis of NbSGT1-HA accumulations in the systemically leaves after transiently expressed in local leaves of *N. benthamiana* plant, using an anti-HA antibody. Procease Ponceau S staining was used to show the protein loadings. Figure S2: Accumulation of TSWV in the TRV infected or non-infected *N. benthamiana* plants. (A) The quantitative RT-PCR analysis of TSWV N expression TRV infected or non-infected plants. The actin was used as the internal reference gene. ** refers to *p* < 0.01. (B) Immunoblot analysis of TSWV N protein accumulation in the local and systemic leaves from TRV infected or non-infected *N. benthamiana* plants. Figure S3: Quantitative RT-PCR analyses of NbSGT1 expression at 14 days after TRV-mediated gene silencing and additional 7 days after tospovirus inoculation. (A and B) Quantitative RT-PCR analysis of NbSGT1 expression silenced by TRV-mediated VIGS in *N. benthamiana* at 14 dpi (A) and at additional 7 days after TSWV infection (B). (C and D) Quantitative RT-PCR analysis of NbSGT1 expression by TRV-mediated gene silencing in *N. benthamiana* at 14 dpi (C) and at additional 7 days after TZSV infection (D). (E and F) Quantitative RT-PCR analysis of NbSGT1 expression by TRV-mediated gene silencing in *N. benthamiana* at 14 dpi (E) and at additional 7 days after INSV infection (F). The actin was used as the internal reference gene. means ± SD, *n* = 4, * refers to *p* < 0.05. Figure S4: Quantitative RT-PCR analyses of tospovirus N gene expression in NbSGT1-silenced or non-silenced *N. benthamiana* plants using EF1a as the internal reference gene. (A) The quantitative RT-PCR analysis of TSWV N expression in the systemically infected leaves harvested from the NbSGT1-silenced or non-silenced plants. The error bars represent SD (*n* = 4), ** refers to *p* < 0.01. (B) The quantitative RT-PCR analysis of TZSV N expression in the systemically infected leaves harvested from the NbSGT1-silenced or non-silenced plants. The error bars represent SD (*n* = 3), * refers to *p* < 0.05. (C) The quantitative RT-PCR analysis of INSV N expression in the systemically infected leaves harvested from the NbSGT1-silenced or non-silenced plants. The error bars represent SD (*n* = 3), * refers to *p* < 0.05.
