# Supplementary material for: Molecular Co-Chaperone SGT1 Is Critical for Cell-to-Cell Movement and Systemic Infection of Tomato Spotted Wilt Virus in Nicotiana benthamiana
Source: Viruses. 2018 Nov 17;10(11):647. doi: 10.3390/v10110647 (PMC6267219; doi:10.3390/v10110647)
Supplement: Supplementary file 1 [file viruses-10-00647-s001.zip › Supplemental files/Supplemental Tables-2018-11-4.docx]

**Supplemental Table S1.** List of primers used in this study.

| **Construct** | **Primers** | **Sequence (5'-3')** | **Purpose** |
| --- | --- | --- | --- |
| p2300S-NbSGT1-3×HA | P1454 | GGGGTACCATGGCGTCCGATCTGGAGAT | To amplify NbSGT1 and clone into p2300S-3×HA. |
|  | P1455 | GCGGATCCGATTTCCCATTTCTTCAGCT |  |
| p2300S-NSm-YFP | P3738 | CGGGATCCATGTTGACTTTTTTTGGTAATAAG | To amplify NSm and clone into p2300S-3×HA. |
|  | P3739 | CGGGATCCTATCTCATCAAAAGATAACTGAGC |  |
| pCV-nYFP-NbSGT1 | Q110 | GGGGTACCATGGCGTCCGATCTGGAGATTAG | To amplify NbSGT1 and clone into pCV-nYFP. |
|  | Q111 | CGGGATCCCTAGATTTCCCATTTCTTCA |  |
| pCV-cYFP-NSm | P4415 | GGGGTACCATGTTGACTTTTTTTGGTAATAAGG | To amplify NSm and clone into pCV-cYFP. |
|  | P4416 | CGGGATCCCTATATCTCATCAAAAGATAACTGAGC |  |
| p2300S-NSm-FLAG | XT654 | CGGGATCCGTATGTTGACTTTTTTTGGTA | To amplify NSm-FLAG and clone into p2300S. |
|  | P1396 | CCGCTCGAGCTACTTATCATCATCATCCTTGTAATCTATCTCATCAAAAGATAACT |  |
| *Nb-EF1a* | P412 | TGCTGCAACAAGATGGATGC | To quantitify the RNA expression of Nb- EF1a . |
|  | P413 | CCAGAGATGGGGACAAAGGG |  |
| *Nb-Actin* | P3433 | GCTCTTGACTATGAGCAGGAGCTTG | To quantitify the RNA expression of Nb-Actin. |
|  | P3434 | GATCATGGATGGCTGGAAGAGGAC |  |
| TSWV-N | Q225 | AGAAGTATGACACCAGGGAAGCC | To quantitify the RNA expression of TSWV-N. |
|  | Q226 | TTGTCTGTTTTTTAACCCCGAAC |  |
| TZSV-N | Q227 | AGATGAGACCACGAAGAAACAAAT | To quantitify the RNA expression of TZSV-N. |
|  | Q228 | AAAGTAAGCCAAGGGAAAGCAAAC |  |
| INSV-N | Q223 | AATCTTGTGTCTTGGGTGTTCTCTTAG | To quantitify the RNA expression of INSV. |
|  | Q224 | GTATATCTGCATATTGTTTTGCCTTAC |  |
| NbSGT1 | Q191 | GGTTGTTTGGGAAGATAACACC | To quantitify the RNA expression of NbSGT1. |
|  | Q192 | ATTTCGAGGAAGGATAACTGGG |  |

**Supplemental Table S2.** Statistic analysis about cell-to-cell movement of NSm.

| Bombarded  plasmid | Host plant | Total foci | Number of cells showing NSm-EGFP signal | | | |
| --- | --- | --- | --- | --- | --- | --- |
|  |  |  | 1 cell 2-4 cells 5≤cells P-value | | | |
| mCherry-HDEL  //NSm-GFP | NbSGT1-Silenced plants | 38 | 29 | 7 | 2 |  |
|  | *N. benthamiana* plants | 36 | 2 | 8 | 26 | ＜0.001 |

P-values were calculated using the unpaired two tailed Student t-test.
