# Supplementary figures and images for: Molecular Co-Chaperone SGT1 Is Critical for Cell-to-Cell Movement and Systemic Infection of Tomato Spotted Wilt Virus in Nicotiana benthamiana
Source: Viruses. 2018 Nov 17;10(11):647. doi: 10.3390/v10110647 (PMC6267219; doi:10.3390/v10110647)

**A**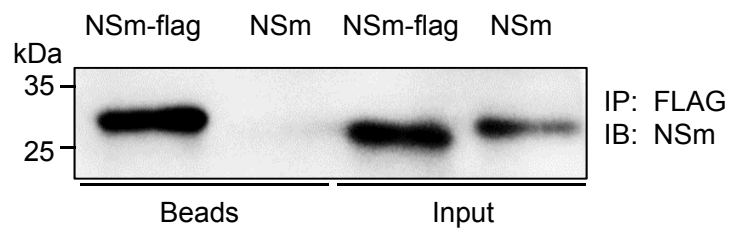**B**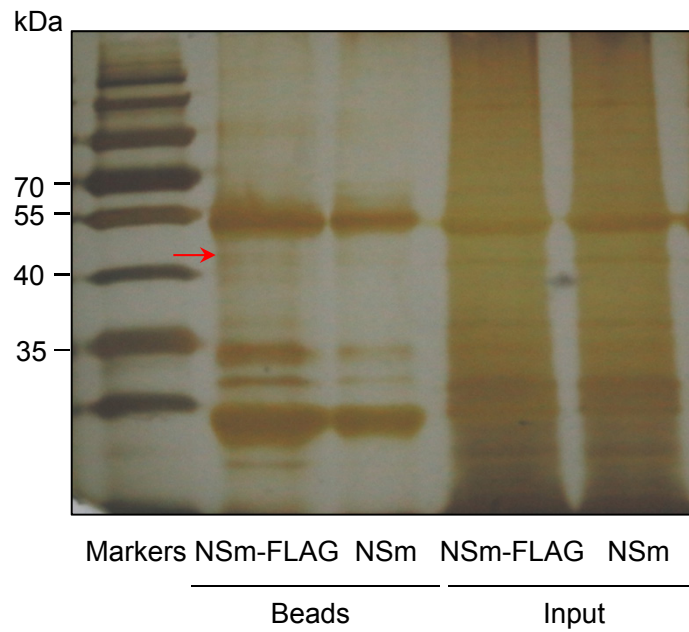**C**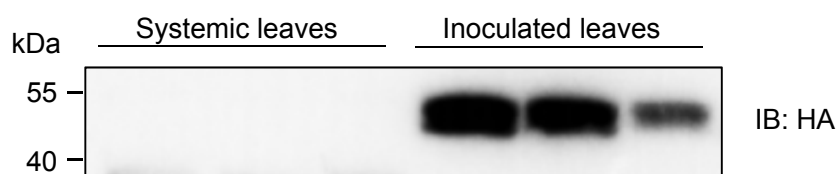**Figure S1.**

**A**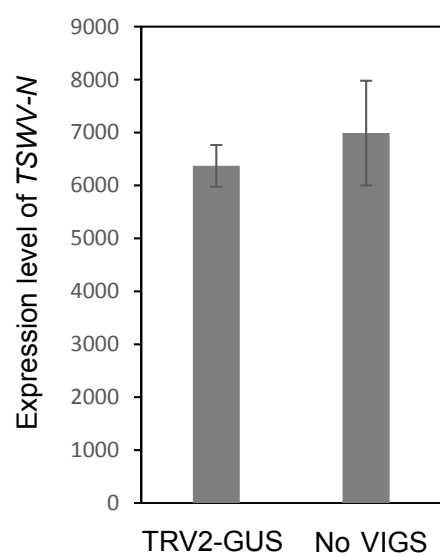**B**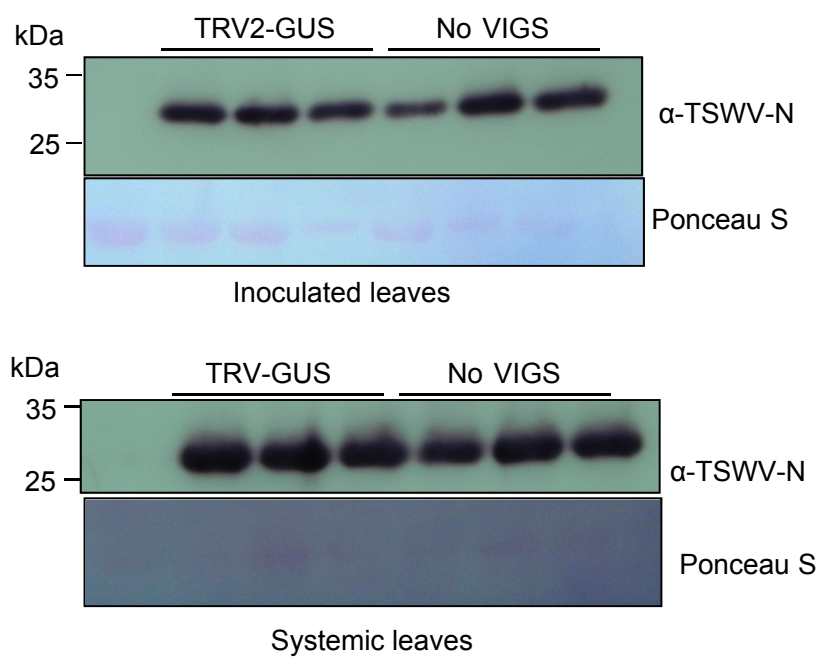**Figure S2.**

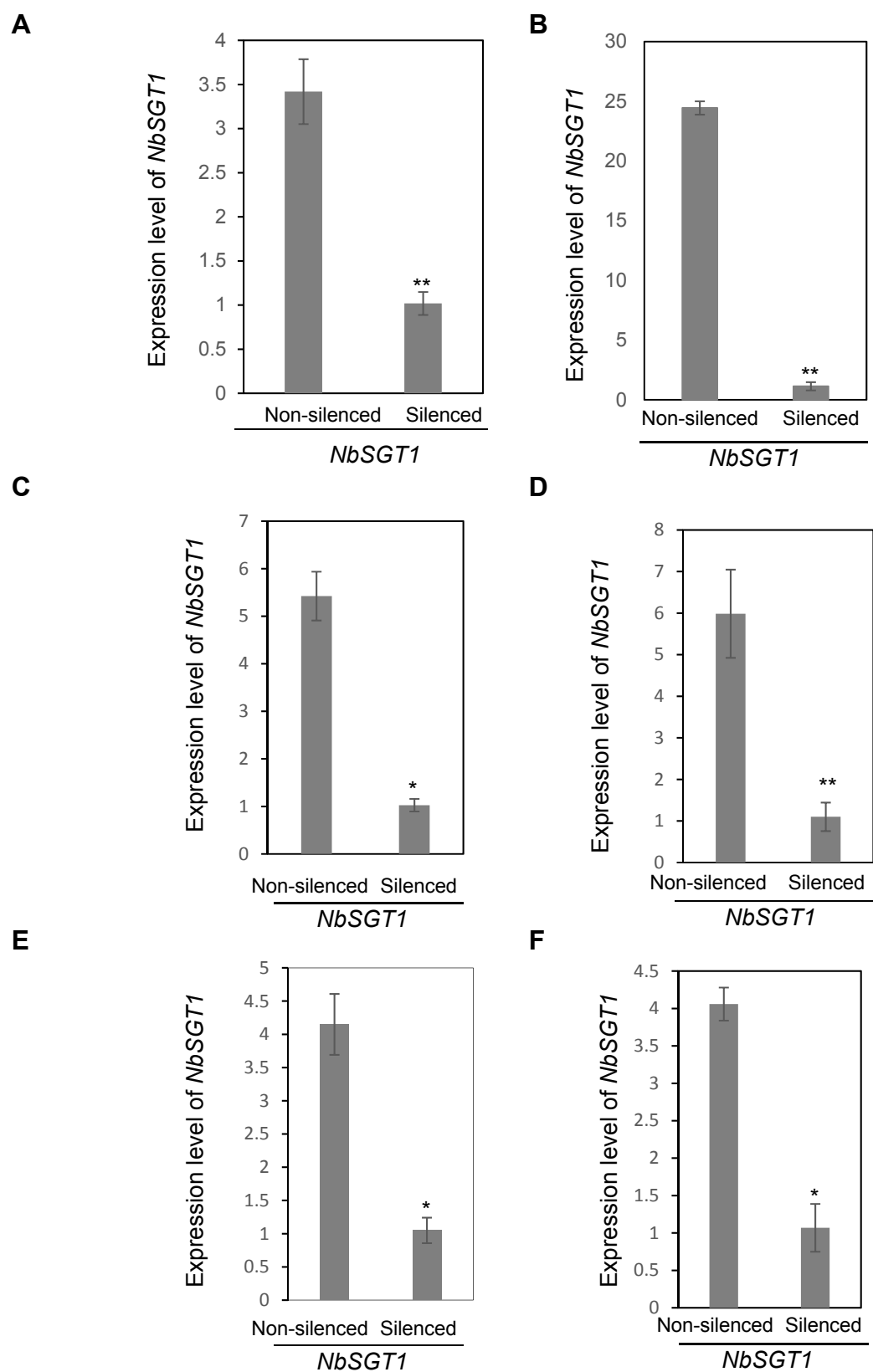

**Figure S3.**

**A**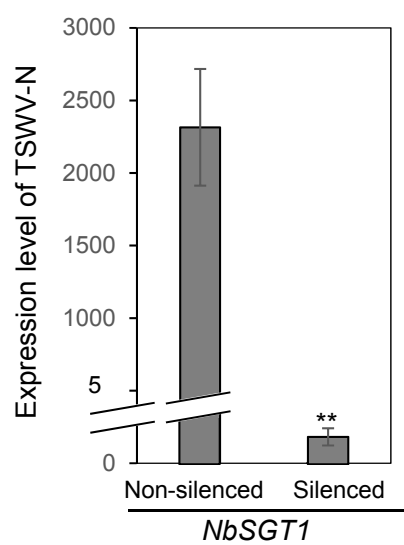**B**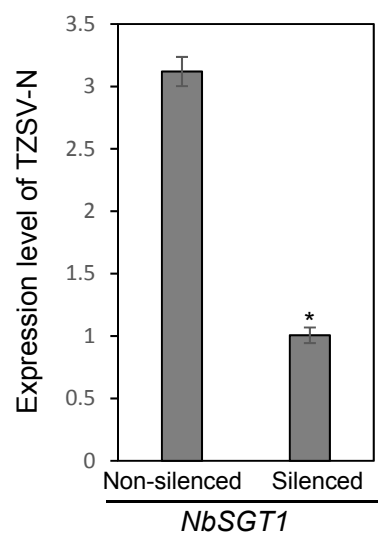**C**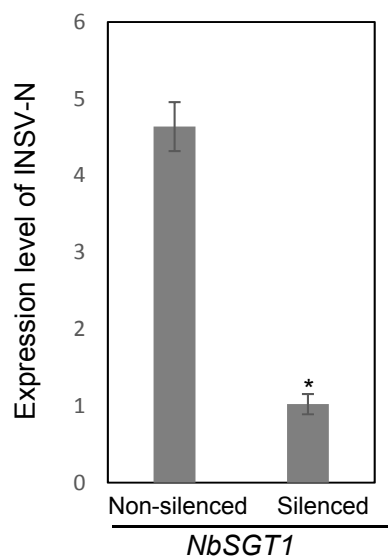

Supplement: Supplementary file 1 [file viruses-10-00647-s001.zip › Supplemental files/Supplemental Figures.pdf]
